# The ‘Male Flower’ of *Ricinus communis* (Euphorbiaceae) Interpreted as a Multi-Flowered Unit

**DOI:** 10.3389/fcell.2020.00313

**Published:** 2020-04-30

**Authors:** Regine Claßen-Bockhoff, Hebert Frankenhäuser

**Affiliations:** Institute of Organismic and Molecular Evolution, Faculty of Biology, Johannes Gutenberg University of Mainz, Mainz, Germany

**Keywords:** development, floral unit meristem, flower-inflorescence boundary, stamen homology, staminal trees

## Abstract

One of the most exciting questions in botany refers to the nature of the angiosperm flower. While most flowering structures are easily identified as flowers, there are few examples lying in-between flowers and inflorescences. Such an example is the staminate unit (‘male flower’) in *Ricinus communis* (Euphorbiaceae) famous for its branched ‘staminal trees.’ The units were controversially interpreted in the past. Today, they are seen as flowers with multiple branched stamen-fascicles. In the present paper, the recently described floral unit meristem is used to reinterpret the staminate units in *Ricinus*. This meristem shares almost all characteristics with a flower meristem, but differs from it in the number of fractionation steps resulting in multi-flowered units. Reinvestigation of the development confirms previous studies illustrating up to six fractionation steps before the meristem merges into anther-formation. Fractionation starts early at a naked meristem, covers simultaneously its whole surface, shows an all-side instead of unidirectional splitting pattern and continues repeatedly. Based on the present knowledge, it is plausible to interpret the ‘male flower’ as a floral unit with multiple staminate flowers each reduced to a single anther. This interpretation is in accordance with the many examples of reduced flowers in the Euphorbiaceae.

## Introduction

One of the most exciting and fundamental questions in botany refers to the nature of the angiosperm flower. In the past, the flower was interpreted as an euanthium, which is a monaxial shoot with modified leaves (von Goethe, 1790; Arber and Parkin, 1907; Glover, 2007; Ronse De Craene, 2010), or as a highly reduced polyaxial system, a pseudanthium (Delpino, 1889, 1890; von Wettstein, 1901–1908 and followers of alternative flower theories). Today, the euanthium theory is widely accepted though many discrepancies still exist (reviewed by Claßen-Bockhoff, 2016).

Conflicts occur when floral organs change position as in the Triuridaceae *Lacandonia* (Rudall, 2003; Ambrose et al., 2006), when floral organs appear in high numbers as in *Centrolepis* (Centrolepidaceae, Sokoloff et al., 2009) and *Tupidanthus* (Araliaceae, Sokoloff et al., 2007) or when flowers are highly reduced and aggregated as in *Euphorbia* (Prenner and Rudall, 2007). In these cases, the term pseudanthium reappears indicating that the morphological nature of the angiosperm flowers is still up for debate.

**FIGURE 1:**
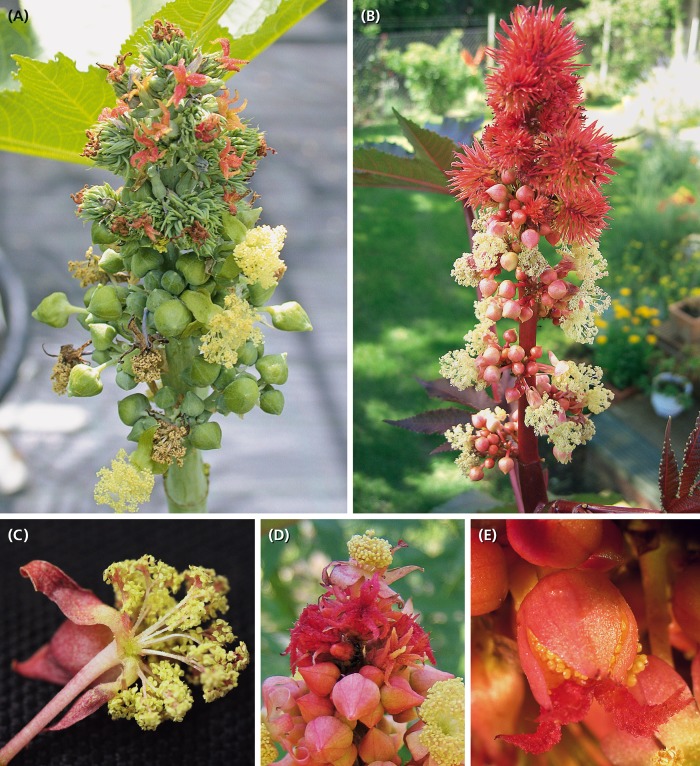
*Ricinus communis*. **(A,B)** Inflorescence in different flowering stages. Carpellate flowers in distal and staminate units in proximal positions. **(C)** Staminate unit composed of five reddish bracts/sepals and about 25 much-branched staminal trees, each branch ending in an anther. **(D)** Rare case of inflorescences with a staminate unit in terminal position. **(E)** Rare case of hermaphrodite units consisting of a gynoecium in the center surrounded by few staminal trees.

An obscure structure is the ‘male flower’ of the castor oil plant *Ricinus communis* L. (Euphorbiaceae). It is characterized by unique ‘staminal trees,’ each bearing several anthers on a branched supporter (Figure 1C). This peculiar structure was interpreted in various ways (reviewed by Prenner et al., 2008). Delpino (1889, 1890) called it a pseudanthium, a term introduced by him to characterize contracted inflorescences (‘infiorescenze contratte’) hardly distinguishable from flowers. Zimmermann (1930) used the structure to extend his telome theory to higher plants, a view followed by Wilson (1942). Lam (1948) noticed the appendages below the anthers and argued that these structures could be the subtending bracts of uni-staminate flowers, consequently interpreting the ‘flower’ as an inflorescence. Since van der Pijl (1952) critically discussed the different views, the staminate units were mainly interpreted as flowers with multiple branched stamen fascicles and connective effigurations.

Prenner et al. (2008) reinvestigated the development of the ‘male flower’ in *Ricinus* to clarify its flower vs. inflorescence nature in a morphogenetic and phylogenetic context. The authors confirmed the repeated meristem fractionation during the process of staminal fascicle formation already documented by Payer (1857) and Michaelis (1924). All authors came to the conclusion that the staminate unit might be rather interpreted as a polyandric flower than an inflorescence.

The difficulty to interpret the unusual structure in *Ricinus* may be intimately connected with the reference system only considering flowers and inflorescences. Consulting the floral unit meristem (FUM), introduced as a third reproductive meristem some years ago (Claßen-Bockhoff and Bull-Hereñu, 2013), may help to disentangle the morphological nature of intermediate structures.

Traditionally, inflorescence and flower meristems are both referred to the shoot apical meristem (SAM). They produce either flower bearing shoot systems (inflorescences) or short shoots with reproductive organs and often preceeding sterile organs (flowers). However, detailed studies on vegetative and reproductive meristems indicate that two different meristems exist producing multi-flowered units, the inflorescence meristem (IM) resembling the SAM and the FUM more similar to a flower meristem (FM) (Claßen-Bockhoff and Bull-Hereñu, 2013; Claßen-Bockhoff, 2016).

• IMs differ from SAMs in having a limited apical growth activity and developing immediately flowers or partial inflorescences from axillary meristems. Only rarely, subtending bracts are lacking. The IMs still produce new primordia in an acropetal sequence and share, thus, the process of segregation with SAMs. Segregation is defined as the lateral separation of meristem parts (primordia) at an ongoing growing meristem (Claßen-Bockhoff and Arndt, 2018).
• FMs differ from SAMs and IMs in being determinate. They lack apical growth activity and are instead able to expand toward different directions. Flower development usually starts with meristem enlargement forming a naked stage which is then used completely by floral organ formation. To distinguish this process from segregation, it is called fractionation (Claßen-Bockhoff, 2016; Claßen-Bockhoff and Arndt, 2018). Fractionation is restricted to determinate meristems, which are much more affected by spatial constraints than segregating meristems.
• FUMs share almost all characteristics with FMs, i.e., they are determinate, able to expand and to use the meristem completely by fractionation. However, they differ from FMs in the number of fractionation steps. In the heads of Asteraceae, for instance, the first fractionation results in the formation of FMs, which, in a second step, gives rise to floral organs. In secondary heads, the first fractionation originates head meristems, the second FMs and the third floral organ primordia (Claßen-Bockhoff, 2016). Subtending bracts are often lacking in floral units.

Beyond Asteraceae, FUMs were already proven in more than 10 angiosperm families (Claßen-Bockhoff and Bull-Hereñu, 2013; Stützel and Trovó, 2013; Naghiloo and Claßen-Bockhoff, 2017; Claßen-Bockhoff and Arndt, 2018). It is, thus, evident that multi-flowered units traditionally summarized as inflorescences fall into two different groups (Bull-Hereñu and Claßen-Bockhoff, 2011).

The present paper reinvestigates the development of the inflorescence, carpellate flowers and staminate units in *R. communis* and tests the hypothesis, that the staminate unit is neither a flower nor an inflorescence, but a floral unit.

## Materials and Methods

*Ricinus* is a monotypic genus most likely originating from N-Africa. The only species, *R. communis* L., produces the castor oil which has been used for 1000s of years (van Welzen, 1998). The plant, cultivated and spread throughout the subtropics and tropics, is morphologically highly diverse. The results of the present study refer to plants cultivated in Germany.

Inflorescence architecture, flower morph distribution and flowering sequence were studied on 13 plants cultivated in private and public gardens around Mainz, Germany. Each plant was observed daily for 14 days and branching pattern and flowering sequence were recorded by notes and photos.

For the morphogenetic analysis, plant material was taken from the Botanical Garden of Mainz University. Inflorescence buds and young inflorescences of different developmental stages were collected and fixed in 70% EtOH. In total, 5 buds and 10 young inflorescences were dissected each providing 20–50 floral meristems of different age. The samples were dehydrated in an alcohol-acetone series, critical point-dried (BAL-TEC CPD030) and mounted and sputter coated with gold (BAL-TEC SCD005). Finally, they were observed and analyzed using the scanning electron microscope (ESEM XL-30 Philips). All steps were conducted according to the manufacturer’s protocol.

To illustrate meristem expansion during the process of repeated fractionation, the diameter of the meristem (without collar) was measured using SEM pictures. At least 10 samples of each fractionation step were included. As the meristem expands hemispherically, the surface plane was calculated using the formula for measuring the surface of a sphere (3,14 x diameter^2^) divided by two. This rough approximation was used to depict meristem expansion rather than to provide reliable quantitative data.

The inflorescence is usually interpreted as being composed of male and female flowers each surrounded by a calyx. As the homology of the staminate unit (male flower) is the topic of the present paper, we use neutral terms.

In the following, we distinguish carpellate flowers with a calyx and staminate units with a collar. We avoid the terms ‘female’ and ‘male’ as they belong to the sexual generation of the gamatophyte. We restrict the term ‘stamen fascicle’ to flowers with secondary polyandry and use the term ‘staminal tree’ as a descriptive term to characterize the unique structure in *Ricinus*.

## Results

**FIGURE 2:**
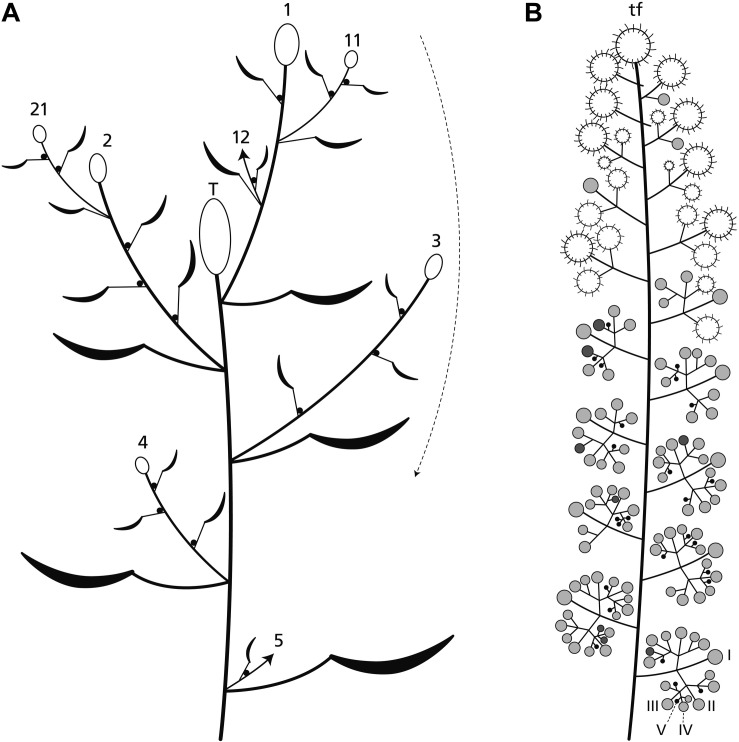
Plant and inflorescence architecture. **(A)** The main axis ends in a terminal inflorescence (T). Lateral axes (1–4) also produce terminal inflorescences which flower in basipetal direction (arrow). Uppermost branches overtop the terminal inflorescence and continue to branch (11, 21) in a sympodial manner. **(B)** The inflorescence is a thyrse with a terminal unit (usually a terminal flower: tf) and up to five branch orders (I–V). Its distal part bears predominantly carpellate flowers (large circles), the proximal one staminate units (small circles). The pictured inflorescence is at the end of anthesis. The carpellate flowers are already fruiting, most of the staminate units are in the postfloral stage (gray) few of them are flowering (dark gray) or still in the bud stage (black).

*Ricinus communis* is a fast-growing herb reaching a height of several meters. The main axis and all lateral axes of first and higher branch orders terminate each in a much-branched, monoecious inflorescence (Figures 1A,B, 2B). The terminal inflorescence (Figure 2A: T) is the largest one and flowers first. It is followed by first order lateral inflorescences, which decrease in size top down and flower in basipetal sequence (Figure 2A; arrow). The distal branches overtop the main axis continuing in a sympodial way. All leaves are large and frondose and produce extrafloral nectaries below the peltate part of the lamina. These nectaries and additional ones at the nodes of the plants attract ants.

### Inflorescence Architecture and Development

**FIGURE 3:**
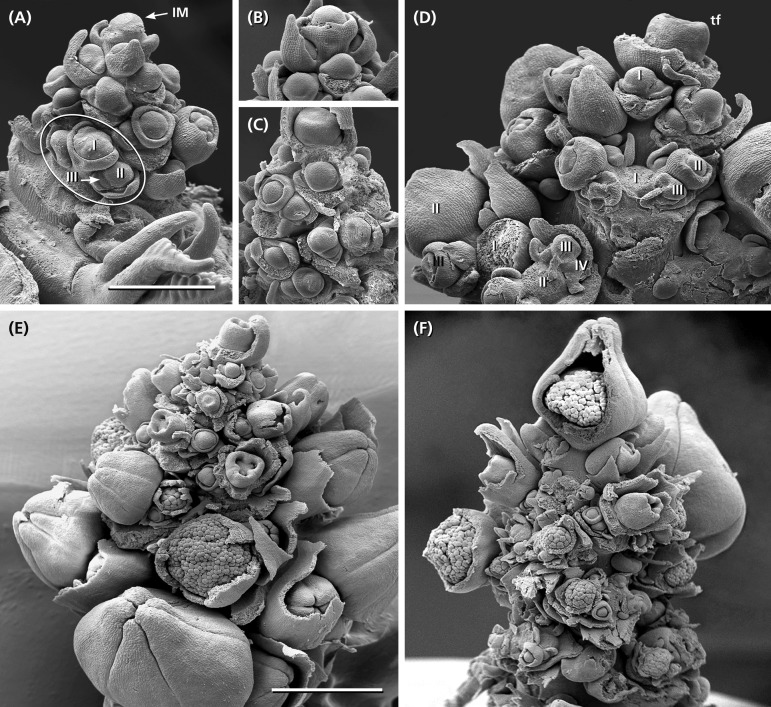
Inflorescence development. **(A)** Young inflorescence with an inflorescence meristem (IM) still segregating bracts and axillary meristems. Developmental sequence is acropetal along the main axis and ordinal within the lateral branches (I, II, III). Cymes start with a regular sympodial-dichasial pattern. **(B,C)** The tip of the IM merges into a carpellate flower surrounded by a synsepalous calyx. **(D)** Detail of a more developed inflorescence already branched up to the forth order (I–IV). Some flowers removed to show the regular dichasial branching pattern. **(E)** Side view of a young inflorescence showing the acropetal development of the lateral branches. In part of the flowers, the outer enveloping elements removed to show the developmental stage of the carpellate flowers and staminate units. **(F)** Developing inflorescence with a terminal staminate unit. Bars: 500 μm **(A)** and 1 mm **(E)**. **(A–D,F)** In the same scale.

The inflorescence is a thyrse with many laterally arranged, almost sessile cymes. It is usually determined by a carpellate flower (Figure 2B: tf) and only rarely by a staminate unit. The proximal cymes are dichasially branched up to the fifth branch order (Figures 2B: I–V, 3D: I–IV), the higher orders sometimes ending monochasially. They bear exclusively staminate units. The tip of the thryrse is usually crowned by a carpellate flower. Only rarely (19%, *n* = 39), a staminate unit occupies the terminal position (Figures 1D, 3F). The distal branches are less branched than the proximal ones and bear carpellate flowers only.

**FIGURE 4:**
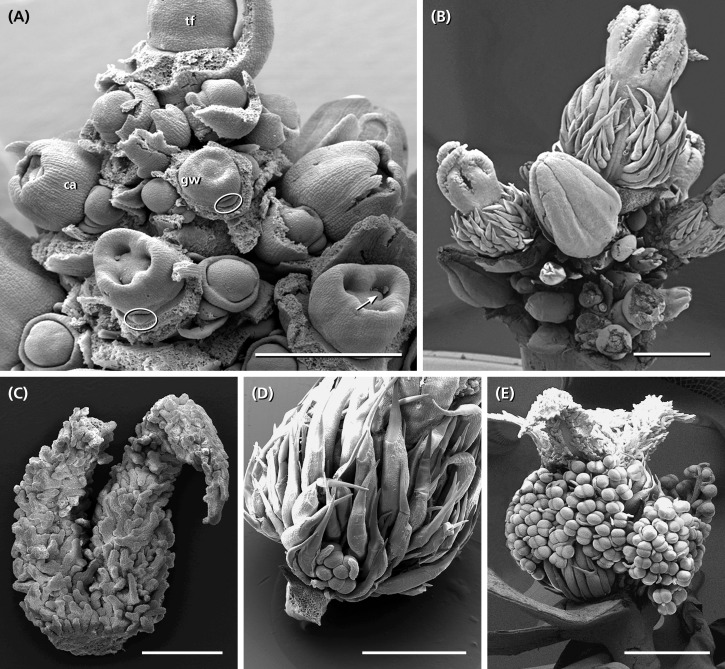
Development of the carpellate flower. **(A)** The developing gynoecium is enclosed by a synsepalous calyx (ca). When the calyx is removed, the formation of a meristematic ring is observable originating the outer gynoecium wall (gw). The ring continues growing, overtops the base of the gynoecium and starts to form the septa of the gynoecium. In each locule, a single ovule appears in axial position (arrow). Quite often, a structure outside the gynoecium appears not further developing (circle). **(B)** The gynoecium of old buds is covered by long hairs and crowned by three bifid styles. **(C)** Bifid style densely covered with papillate outgrowths becoming sticky in the mature stage. **(D,E)** Rare cases of hermaphrodite units with few to many staminal trees surrounding the central gynoecium. Bars: 200 μm **(A)**, 500 μm **(C)**, 1 mm **(B,D)**, 2 mm **(E)**.

Between the two unisexual zones, an intermediate zone appears bearing carpellate flowers and staminate units in mixed patterns (Figure 2B). On average, a single terminal inflorescence produces 15–21 carpellate flowers and 130–140 staminate units (*n* = 5) illustrating that there are about 7.5-times more staminate units than carpellate flowers. However, the general pattern is not strictly fixed as to the size of the inflorescence and the relative number of carpellate flowers vs. staminate units. In one case, an inflorescence was predominantly carpellate producing carpellate flowers even in the proximal part. As further exceptions, hermaphrodite units were found (Figures 1E, 4D,E) occupying the terminal position or the first order position in the intermediate zone.

The inflorescence develops from an inflorescence meristem. It segregates lateral units in an acropetal order each composed of a bract and its axillary meristem (Figure 3A: IM). The axillary meristems produce the cymes. While the tip of the young inflorescence still segregates new units, the axillary meristems of the proximal bracts start to initiate meristems of 2nd and 3rd order (Figure 3A: I–III). They immediately subdivide into two transversely arranged prophylls and a central primordium giving rise to either a carpellate flower or staminate unit. The central primordium develops a ring-like bulge originating the calyx (carpellate flower) or collar (staminate unit), respectively (Figures 3A,B). This structure grows very fast and has to be removed to observe further meristem development.

The terminal unit of the inflorescence, once formed, develops faster than the uppermost lateral flowers (Figures 3B,C). The lateral cymes consecutively produce primordia (Figure 3D: I–IV) in a sympodial-dichasial manner presenting different developmental stages at the same time (Figure 3E).

### Development of Carpellate Flowers

The carpellate flower consists of a single trimerous gynoecium surrounded by a synsepalous calyx. In rare cases, single or many staminal trees appear outside the gynoecium altering the carpellate flower into a hermaphrodite unit (Figures 4D,E).

The meristem of a young carpellate flower is dome-shaped (Figure 3B). The pentamerous calyx early develops and envelops the remaining naked meristem (Figures 3C, 4A: ca). This meristem forms a ring-like bulge which gives rise to the gynoecium wall (Figure 4A: gw). Further development involves the continuous growth of this wall and the formation of three septa subdividing the gynoecium into three locules. In each locule, a single ovule appears in axial position (Figure 4A: arrow). At an older stage, the three carpel lobes give rise to three bifurcate styles (Figure 4B). Their inner side is covered with papillate outgrowths enlarging the surface considerably (Figure 4C). During anthesis, the stylar arms are reddish and spread backward (Figure 1A); the papillate surface gets sticky and catches pollen grains. In old buds, the gynoecium is covered by long hairs which are greenish during anthesis (Figure 1A). In the fruiting stage they become rather long, red and stiff providing the young fruits with their characteristic prickly look (Figure 1B).

At the base of young gynoecia bulges appear which do not develop anymore (Figure 4A: circles). However, as to their position they may have the potential to originate staminal trees.

### Development of Staminate Units and Staminal Trees

**FIGURE 5:**
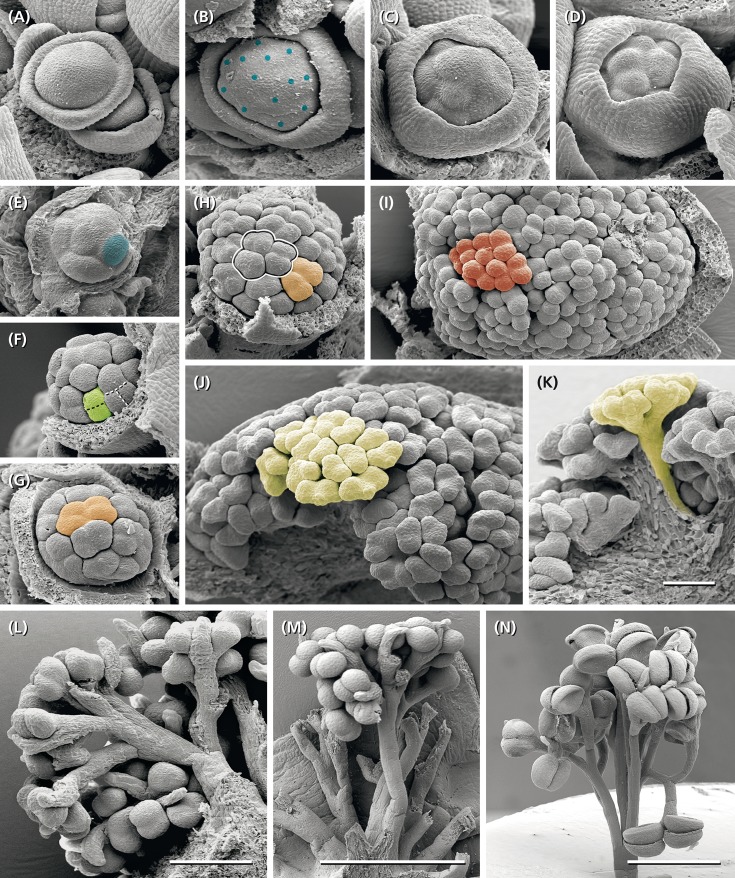
Development of the staminate unit. **(A)** Young dome-shaped primordium with a peripheral, weakly five-lobed meristematic ring. **(B)** Collar clearly separated from central meristem bulge (diameter ∼ 215 μm). **(C,D)** Meristem enveloped by the collar starts first fractionation (diameter ∼ 250 μm). **(E)** Same stage as in **(C)** but smaller (∼165 μm); collar removed, one of the eight submeristems indicated by blue color. **(F)** Second fractionation starting at the peripheral submeristems (green). **(G,H)** Third fractionation at different meristem sizes producing four submeristems (orange) from one original submeristem (blue in **E**). **(I)** Fourth fractionation completed (red, diameter ∼600 μm), begin of staminal tree elongation. **(J,K)** Anther formation after the fifth step of fractionation. **(L–N)** Staminal trees on different developmental stages; the bifurcate splits indicating the consecutive steps of fractionation. Colors indicate the fate of a single original submeristem. **(A–K)** All pictures in the same scale. Bar: 100 μm. Bars in **(L)**: 200 μm, in **(M,N)**: 1 mm.

**FIGURE 6:**
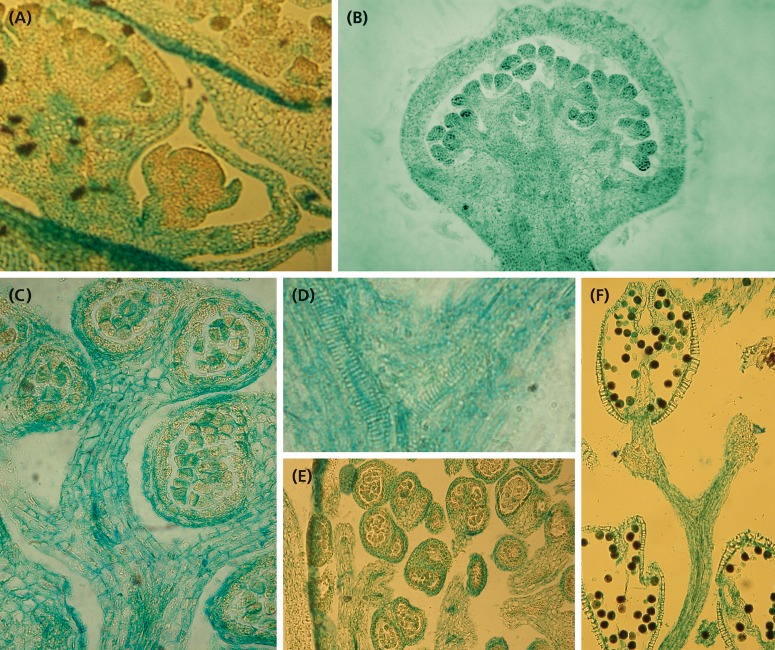
Histology of developing staminate units. **(A)** Longitudinal section of a cyme with two staminate units in different developmental stages. The youngest one corresponds to Figure 5A, the second one to Figure 5H. **(B)** Longitudinal section of a staminate unit enveloped by the collar. **(C)** Longitudinal section of a stalked, almost mature anther. **(D)** Longitudinal section of the upperpart of a stalk segment showing the splitting vascular bundles. **(E)** Cross section of an old staminate bud showing bithectae anthers and vascularized anther stalks. **(F)** Upper part of a staminal tree showing the split of the fourth and fifth fractionation and one mature theca with naked endothecium and pollen grains.

**FIGURE 7:**
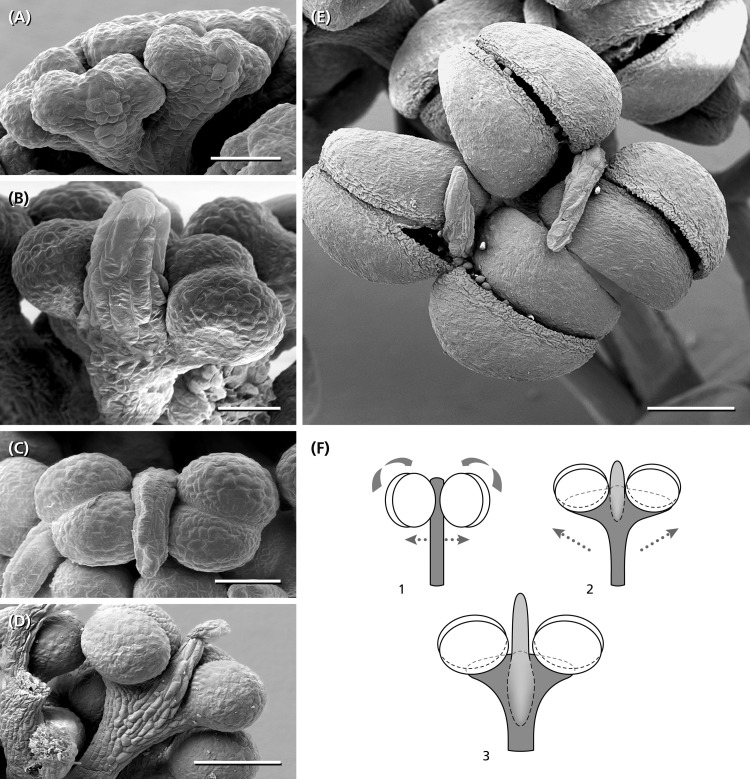
Anther development. **(A)** Young bithectae anthers after the fifth step of fractionation, each with a weak effiguration at the abaxial side. **(B)** Effiguration overtops the two thecae each of them divided into two pollen-sacs. **(C,D)** Anther and abaxial effiguration from above and from the side. **(E)** Two anthers from above presenting their longitudinal dehiscence slits. **(F)** Schematic reconstruction of anther fomation. Compared to a typical bithecate angiosperm anther (*1*), the young anther in *Ricinus* (*2*) shows a widening of the connective turning the thecae around 90° (arrows). At the abaxial side below each theca, an effiguration develops, which in the mature stage (*3*) overtops the pollen-sacs. *1*, angiosperm stamen as reference. *2*, young anther stage corresponding to **(B)**. *(3)* Almost mature stage as shown in **(D)**. Bars: 50 μm **(A,B)**, 100 μm **(C)**, 200 μm **(D,E)**.

Staminate units (‘male flowers’) consist of 20–26 staminal trees surrounded by five sepal-like structures (Figure 1C), which are basally fused (Figures 5A–D). Each staminal tree produces up to 14 bithecate anthers resulting in a total number of 200–350 anthers per staminate unit. Anthers are arranged at the end of bifurcate stalks (Figures 5L, 6C,F) and always associated with a bract-like structure at their abaxial side (Figure 7D).

The meristems of the staminate units resemble carpellate flower meristems. They initially produce a ring-shaped, weakly five-lobed bulge (Figure 5A). This bulge, which later gives rise to the sepal-like structures, quickly develops and envelops the expanding, still naked center of the meristem (Figure 5B).

• In a first step, the center fractionates 8–13 (rarely more) sub-meristems (Figures 5B,E: blue) which arch upward (Figures 6A). Dependent on their position close to the periphery or in the center of the original meristem, they have an oval or roundish shape (Figure 5E).
• In a second step, each sub-meristem of 1st order splits into two (rarely three) equal sub-meristems of 2nd order (Figure 5F: green). These sub-meristems are the initials of the 20–26 staminal trees.
• With the expanding meristem (Table 1), each of the initials passes up to four further synchronous, occasionally imperfect steps of fractionation generating an average of 14 anthers.

**TABLE 1:** Meristem expansion during the process of repeated fractionation (*n* = 10, except the two latest stages).

Developmental steps	Meristem diameter [μm]	Meristem surface [μm^2^]	Degree of surface expansion		
			x-Times	Factor	
Original meristem	177.1 ± 13.93	98,484.25			
1st fractionation (blue)	206.4 ± 20.43	133,767.01	1,358	1,358	1.34± 0.13
2nd fractionation (green)	251.5 ± 18.81	198,612.07	2,016	1,485	
3rd fractionation (orange)	292.8 ± 19.84	269,197.98	2,733	1,355	
4th fractionation (…)	315.7 ± 25.30	312,952.78	3,177	1,163	
5th fractionation (red)	∼600	1130,400.00	11,478	3,612	
6th fractionation (yellow)	∼930	2715,786.00	27,576	2,403	

*x-Times: multiplication of the original meristem surface. Factor: enlargement factor between the single steps of fractionation.*

The size of the original meristem and the number of fractions vary considerably (compare Figures 5B and E and Table 1). However, as a general rule, a sub-meristem always splits at right angle to the previous fractionation resulting in a crosswise alternation of splitting directions. Fractionation happens almost simultaneously covering the whole surface of the meristem. In the beginning some meristems show an advanced fractionation activity at the periphery (Figure 5F). Interestingly, the fractions are of almost similar size (Figures 5E–H) indicating that the meristem surface continuously expands (Table 1). Expansion leads to the hemispherical shape of the developing unit and appears to be quite constant. Roughly estimated, the meristem surface expands 1.34-times with each of the first four steps of fractionation reaching a threefold enlargement in total (Table 1). After the fourth split, the fractionation processes cannot be followed in detail any longer (Figures 5I,J). The fractions remain smaller (Figure 5I) and are elevated in groups by the elongating base of the developing staminal tree (Figures 5K, 6B). Thereby, the surface expands significantly generating space for the development of 100s of anthers (Table 1). Irregularities in the splitting behavior cause the variable number of anthers per staminal tree.

### Formation of Anthers

The last step of fractionation goes along with anther formation (Figure 5J). The young anther has a triangular shape and consists of two premature thecae and an abaxially arranged sterile structure (Figure 7A). When fractionation is regular, two anthers are arranged in pairs looking like mirror images or single anthers appear (Figure 5J). Usually, 12–14 (10–16) anthers are elevated as a group and form a single tree-like bundle (Figures 5L–N). The peculiar feature of this staminal tree is its repetitive branching reflecting the successive steps of fractionation by bifurcate segments. Anthers are placed at the ends of the last segments (Figures 6B,C). From the very first, thecae are oriented horizontally presenting their longitudinal slit in an upward direction (Figures 7A,B,E,F). The unusual position is made available by a broad connective which also clearly separates the thecae from each other. Within the pollen sacs, pollen mother cells develop passing meiosis and producing pollen grains (Figures 6C,E,F). In the mature stage of theca development, the epidermis becomes reduced and the endothecium is exhibited as the outermost cell layer (Figure 6F).

Each staminal tree is provided with a vascular bundle branching at the bifurcation sites and supporting the anthers (Figures 6C,D). In contrast, the abaxial structure which reaches a final length of 0.2 mm, has no vascular bundle. It elongates, gets a flat, bract-like shape, overtops the developing thecae (Figures 7B–D) and finally desiccates (Figure 7E). Epidermis cells of the structure clearly differ from the sculptured thecae walls indicating that the structure originates below the thecae (Figures 7A,D,F).

## Discussion

For more than 100 years, the staminate unit of *R. communis* has fascinated botanists and provoked controversial interpretations. Prenner et al. (2008) summarized this history and discussed whether the *Ricinus* stamen could represent a reduced flower as in several other Euphorbiaceae that possess an obscure flower-inflorescence boundary, but finally concluded that the staminal trees should be taken as extended stamen fascicles.

While intermediate structures or even ‘hybrids’ between flowers and inflorescences are widely accepted (e.g., Prenner and Rudall, 2007; Kirchoff et al., 2008), their morphogenetic and phylogenetic significance is not yet fully understood. Gene expression patterns are used to differentiate among flowers and inflorescences. Prenner et al. (2011) found expression of the LFY protein not only in individual flower primordia of *Euphorbia*, but also in the cyathium primordium indicating that pseudanthial meristems may be genetically similarly regulated like flower meristems. Likewise, Zhao et al. (2016) identified a *Gerbera* specific *GhLFY* resembling *LFY* expression in a single flower meristem. Floral identity and floral symmetry genes were furthermore identified in the showy leaves of *Davidia involucrata* (Vekemans et al., 2012) and showy branchlets in *Actinodium cunninghamii* (Claßen-Bockhoff et al., 2013). All these examples illustrate that one should be careful using gene expression patterns for floral organ homology (Ronse De Craene, 2007; Ochoterena et al., 2019).

Considering the clear differences between inflorescence and flower meristems (summarized in Claßen-Bockhoff, 2016), the question rises whether an extended reference system including floral units could be better suited to interpret ‘intermediate structures.’

### Repeated Fractionation and Spatial Constraints in *Ricinus communis*

The re-investigation of reproductive units in *R. communis* confirms the ontogenetic studies of the ‘male flower’ by Payer (1857); Michaelis (1924), Kirchoff et al. (2008), and Prenner et al. (2008) showing several steps of meristem fractionation before anthers appear. However, also differences were found questioning the given morphological interpretation.

With respect to Prenner et al. (2008) neither the centrifugal development of the first primordia nor the terminal position of one of the staminal trees and the separate initiation of the stamen fascicles are confirmed. Instead, a pattern of meristem fractionation is documented indicating that the availability of space predominantly guides pattern formation.

The first step of meristem development is an almost simultaneous division into sub-meristems of similar size. Dependent on the variable size of the initial meristem, the number of sub-meristems varies. Thereby, neither a terminal sub-meristem nor a flower-characteristic phyllotaxis is observed. Both findings characterize the process of fractionation as a self-organizing process using the meristem completely (Hernandez and Palmer, 1988; Reinhardt et al., 2003; Prusinkiewicz and Barbier de Reuille, 2010; Claßen-Bockhoff and Meyer, 2015). In the next step, the fractions start to grow. Thereby, some sub-meristems show a more advanced development at the periphery indicating that space and shape of the initial meristem might affect the promotion of individual sub-meristems.

Spatial conditions also influence the ongoing meristem development characterized by repetitive fractionation. The initial meristem continuously expands and thereby generates new space for the developing sub-meristems. During the first four steps of fractionation, the degree of expansion is correlated with the temporal sequence of splitting. The sub-meristems enlarge to a certain size and get subdivided into further sub-meristems which repeat the process. However, when space becomes more and more limited, irregular fractionations increase, cause the suppression of further splits and result in a variable number of anthers per fascicle.

On average, each staminate unit has 25 staminal trees originating from 8 to 13 sub-meristems. Though the absolute number varies among staminate units, it is evident, that the staminal trees are not initiated separately at the initial meristem, but at least in pairs at the first sub-meristems.

### Homology of Stamens and Stamen Fascicles – The Flower Perspective

**FIGURE 8:**
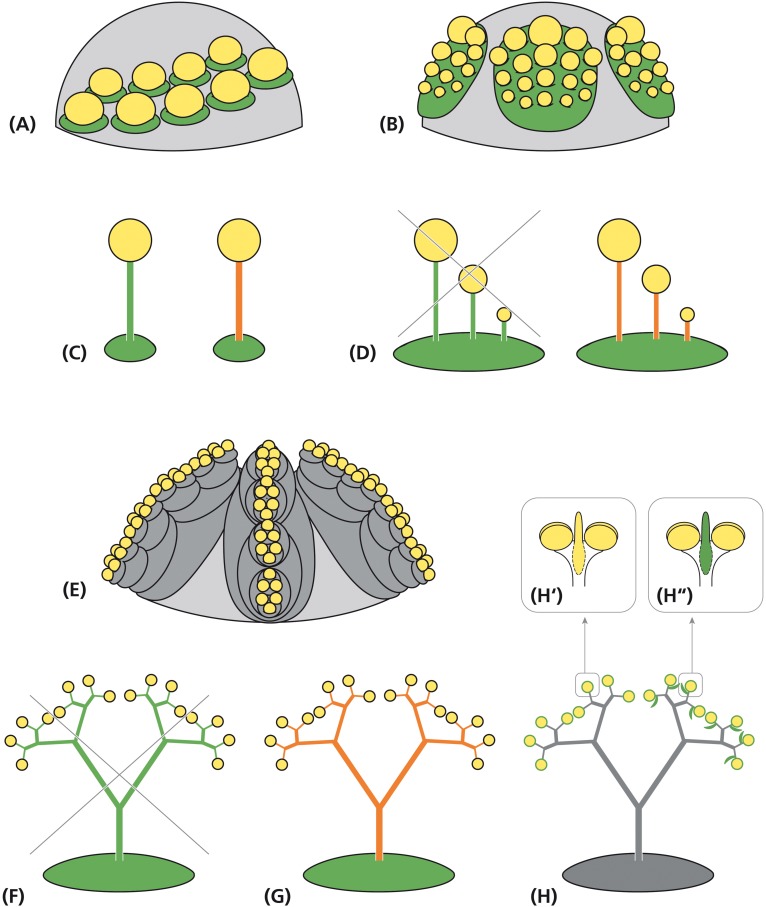
Alternative interpretations of the staminal trees in *Ricinus*. Schematic representation of flower meristems originating single stamens **(A)** and stamen fascicles of the centrifugal type **(B)** from a leaf-homolog primordium (green). **(C)** Morphological interpretation of the filament as part of a sporangiate leaf (left) or an anther supporter (right). **(D)** Sketch of a stamen fascicle interpreted as a compound leaf (left: not confirmed by development) and as a suppressed leaf primordium with stalked anthers (right: uncommon, but acceptable assumption). **(E)** Alternative interpretation: floral unit meristem passing repeated steps of fractionation (gray) before originating reduced, uni-staminate flowers. Interpretations of the staminal tree as **(F)** a stamen fascicle homologous to a compound leaf (not confirmed by development), **(G)** a single stamen with branched anther supporters or **(H)** a floral unit with many uni-staminate flowers arising at the end of bifurcated staminal trees. **(H″′)** Alternative interpretations of the adaxial structure as a connective effiguration (**H′**: yellow) and as a subtending bract (**H″**: green). Green: leaf homolog. Yellow: anther. Orange: effiguration. Gray: meristematic tissue.

Stamen fascicles appear in many plant taxa across the angiosperms (Ronse De Craene, 2010). They are defined as groups of stamens originating from a common primordium by a splitting process (Ronse De Craene and Smets, 1987, 1992). Thereby, the number of stamens increases resulting in a polyandrous androecium (Figure 8B). More specifically, the androecium is called secondary (or complex) polyandrous as a high number of stamens can also arise from many single primordia (primary polyandry, Figure 8A). Usually, the common primordia occupy the position of stamens and are arranged in one or two whorls (Ronse De Craene, 2010). The splitting process proceeds top-down and is centrifugal or centripetal depending on the shape of the flower primordium, local meristem expansion and spatial constraints (Ronse De Craene and Smets, 1991). Secondary polyandry can also arise from a ring primordium and/or proceed in lateral direction, but these forms are not relevant in the present study.

Staminal tree formation in *Ricinus* corresponds with that in secondarily polyandrous flowers in increasing the number of stamens by fractionation. However, neither the position of the fractions nor the direction of splitting conforms to secondary polyandry. Furthermore, the high number of successive steps of subdivision resulting in multiple branched staminal trees is almost unique in *Ricinus*. One may accept these peculiarities as a specific mode of stamen fascicle formation in *R. communis* arguing that the flower meristem is sufficiently plastic to allow this variation. Compared to all other vegetative and reproductive meristems, the FM indeed is the most dynamic one (Ronse De Craene, 2010; Claßen-Bockhoff, 2016). However, interpreting the staminate unit in *Ricinus* as a flower has deep implications on the interpretation of the flower and particularly on the stamen.

While the pollen-sacs correspond to microsporangia arranged in an anther, the question raises what the filament might be? Is it the petiole of a leaf (Figure 8C: green; Baum and Leinfellner, 1953; Kunze, 1979) or is it just an anther supporter (antherophor) elevating the pollen-sacs in a position for optimal pollen dispersion (Figure 8C: orange; Hagemann, 1984)? In this case, to keep the euanthial theory, one could argue that the common primordium is a leaf homolog, while the filament-anther complex is an emergence without being homologous to any part of the leaf. It develops from the surface of a leaf primordium which itself remains inhibited. The emergence hypothesis is attractive as it could easily explain the high structural diversity of the filaments without the need to refer to leaf development. However, considering the deep differences between SAMs and FMs, one might also question the euanthial theory and prefer interpreting the flower just as the tip of the stem bearing floral organs (Claßen-Bockhoff, 2016). As floral organs originate from a determinate FM genetically completely differently regulated than SAMs, there is no need to homologize the flower with a short shoot any longer.

The petiole interpretation of the filament is in conflict with the phenomenon of secondary polyandry where many filaments originate from a single primordium. To solve this problem, Guédès (1979), as Payer (1857) and others before him (summarized in von Wettstein, 1901–1908), compared the branched stamen fascicle with a multi-pinnate leaf (Figures 8D,F: green). This comparison, however, cannot be hold as pinnae originate from the fractionating leaf margin and not from the primordium plain (Hagemann, 1970). Only the emergence interpretation of the filament is in accordance with the phenomenon of secondary polyandry. Accepting this view, the angiosperm filament is an anther supporter. Stamen fascicles only differ quantitatively from solitary stamens as they produce several instead of only one antherophor per underlying primordium (Figure 8D: orange). If the staminate unit in *R. communis* is referred to an euanthium, the collar should be homologized with the calyx of the carpellate flower and the stamen fascicle interpreted as a multiple branched emergence bearing anthers (Figures 8F,G).

### Homology of the Staminal Trees – The Floral Unit Perspective

Multi-flowered units are traditionally called inflorescences. Dependent on the absence or presence of a terminal flower, they are grouped into open and closed inflorescences. In both cases, it is assumed that they develop from IMs sharing many characteristics with SAMs (Prusinkiewicz et al., 2007). However, morphogenetic studies clearly illustrate, that multi-flowered units develop from two different meristems, the IMs generating inflorescences by the process of segregation and FUMs giving rise to floral units (Claßen-Bockhoff and Bull-Hereñu, 2013). FUMs share almost all characteristics with FMs, in particular the determinate nature of the meristem, the initially naked stage and the capacities of expansion and fractionation. For that reason, young FUMs cannot be distinguished from FMs (Claßen-Bockhoff, 2016). The notion of Prenner et al. (2008) that the young stages of the staminate unit meristems in *R. communis* look flower-like is, thus, not necessarily an indication for the flower nature of these units. FUMs differ from FMs in the number of fractionation steps (Figure 8E). As the high number of consecutive meristem splits is the most peculiar feature in the development of the staminal trees, FUMs appear to be excellent candidates to explain the staminate unit in *R. communis*.

Interpreting the staminate unit in *R. communis* as a floral unit means at the same time that the staminal trees are not homologous to the stamen fascicles of polyandrous flowers but to clusters of highly reduced flowers (Figure 8H). Each anther is homologized with a uni-staminate flower. This flower has neither a pedicel nor a filament, but only a well-developed anther, directly originating from the latest sub-meristem.

The anthers may or may not be associated with laterally arranged sterile structures (van der Pijl, 1952). The structure is traditionally taken as a connective effiguration (Figure 8H′), but this interpretation is questionable. First, is it difficult to identify the connective due to the upturned position of the anther. Second, the structure appears to be inserted below the anther directly originating from the sub-meristem. As an alternative, the sterile structure can be taken as the subtending bract of the uni-staminate flower (Figure 8H″, Lam, 1948). The position supports this view and the minute length of the bract could explain the lack of a vascular bundle.

The given scenario of the staminate unit development in *Ricinus* corresponds to the present knowledge about FUMs. It only differs from the known examples in the high number of fractionation steps and in the extreme flower reduction to a single anther. All other floral units investigated so far are clearly composed of flowers and have been recognized as inflorescences in the past. An almost simultaneous subdivision of a large hemispherical meristem similar to that in *Riciuns* was observed in the head development of the dove tree *Davidia involucrata* (Nyssaceae; Claßen-Bockhoff and Arndt, 2018). The meristem likewise expands considerably originating new FMs in basipetal direction (Jerominek et al., 2014). After the first step of fractionation, the sub-meristems merge into FMs. Ongoing meristem expansion enlarges the already existing FMs providing them with the space for floral organ formation. In the secondary heads of *Echinops* and *Pycnosurus* (both Asteraceae; Claßen-Bockhoff, 2016), the compound umbels of Apiaceae (Claßen-Bockhoff and Bull-Hereñu, 2013, unpublished data) and the secondary heads of some Eriocaulaceae (Stützel and Trovó, 2013), floral organs are only formed after a second step of fractionation.

It should not be concealed that the inflorescences of some Urticaceae (*Dorstenia*) and Moraceae (*Elatostema sessilis*, *Procris frutescens*) show a high developmental similarity to *Ricinus* (Bernbeck, 1932). In the mentioned Moraceae, development starts with a large, naked meristem which splits crosswise as in *Ricinus*. The splits are associated with bract formation at the margin of the flat meristem, but flowers are only initiated after the fourth fractionation step. The inflorescences are traditionally interpreted as condensed thyrses with dichasial cymes. A re-investigation is needed to elucidate whether the reproductive systems are inflorescences or floral units. In any case, they support the view that the staminate unit in *Ricinus* is rather a multi-flowered unit than a flower.

### Evolution of Staminate Units in *R. communis*

There is a general agreement that the staminate units of *R. communis* evolved in adaptation to wind pollination (Delpino, 1889; van der Pijl, 1952). Dusty pollen, exposed anthers, papillate and sticky stigmas and lacking perianth structures are characteristic features of anemophilous blossoms. In *R. communis*, pollen grains are additionally released explosively (Bianchini and Pacini, 1996). The specific release mechanism is associated with the loss of the epidermis of the thecae walls (Staedtler, 1923; Bianchini and Pacini, 1996).

The shift from zoophily to anemophily is often associated with extreme flower reduction resulting in perianth-less unisexual flowers which are aggregated in dense clusters of monoecious and dioecious inflorescences (Friedman and Barrett, 2009 and literature herein). All these characters fit to the interpretation that the staminate unit in *R. communis* is not a flower but a floral unit.

The derived character of the staminate units also fits to the phylogenetic relationship of the species. The monotypic *Ricinus* is placed in subclade A4 of the core acalyphoids (Euphorbiaceae s.str., Wurdack et al., 2005). It is classified, though weakly supported, as sister to *Speranskia.* This genus differs considerably from *Ricinus* notably in the presence of 10–15 free stamens per staminate flower indicating that the formation of staminal trees is an apomorphy for *Ricinus*. On the other hand, *Ricinus*-like staminal trees appear in the only distantly related genera *Homonoia* and *Spathiostemon* (van Welzen, 1998) placed in the Acalypheae-Lasiococcinae, subclade A3 (Wurdack et al., 2005). Further examples in Euphorbiaceae (Michaelis, 1924) indicate that peculiar stamen structures evolved several times independently within the family. Unfortunately, these species are only little known and it is completely unclear whether their ‘male flowers’ develop like the staminate units in *Ricinus* or differently. Much better investigated are the cyathia in *Euphorbia* (Prenner and Rudall, 2007) originally interpreted as flowers and then identified as a cluster of unisexual flowers (Čelakovský, 1872; Michaelis, 1924). The terminal position is occupied by a carpellate flower only consisting of a trimerous gynoecium. This flower is surrounded by five groups of staminate flowers each reduced to a single stamen. If the staminate unit in *Ricinus* is interpreted as a floral unit, similarities to *Euphorbia* cannot be denied.

An interesting finding is the appearance of bisexual units in *R. communis* already described by Michaelis (1924) and Prenner et al. (2008). Interestingly, Michaelis (1924) found them also in *Homonoia*, the genus sharing staminal trees with *Ricinus*. As the family is characterized by unisexual flowers, these abnormalities can be either interpreted as rarely appearing perfect flowers or as floral units composed of a single carpellate flower surrounded by several staminate units. Such patterns are known from the floral units in *Davidia involucrata* (Nyssaceae, Claßen-Bockhoff and Arndt, 2018) and from the cyathia in *Euphorbia*.

While cyathia are generally bisexual, both loss of the terminal flower (Narbona et al., 2002; Schardt and Claßen-Bockhoff, 2013) and transition to dioecy result in unisexual cyathia. In *E. balsamifera* and other diecious species, the carpellate cyathium is reduced to a single trimerous gynoecium surrounded by an involucrum bearing the nectaries. Referring to the cyathium, nobody would call this involucrum a calyx. In *R. communis*, the opposite case the obvious. Referring to the assumed flower, the structure surrounding the naked gynoecium is interpreted as a calyx. However, as carpellate and staminate units in *Ricinus* share the same early developmental stages including the naked stage, meristem expansion and calyx/collar formation, it cannot be excluded that the ‘carpellate flower’ in *Ricinus* is also a reduced floral unit. Similar to diecious cyathia, both carpellate and staminate units in *Ricinus* would then be floral units surrounded by a collar.

## Conclusion

In the present paper, the staminate unit in *Ricinus* is interpreted as a floral unit composed of highly reduced uni-staminate flowers. The main argument is the high number of fractionation steps, which run almost simultaneously across the whole meristem surface and show a crosswise splitting pattern. These features are not known from secondarily polyandrous flowers. If the staminate unit is nevertheless interpreted as a flower (which is possible), one has to accept a completely new stamen structure and to disregard the meristem similarities between *R. communis* and floral units. Maybe, it is too early for a final morphological interpretation of the staminate units in *Ricinus* as there is too little known about FUMs, their diversity and genetic regulation. In this sense, the present paper and the following attempt to explain the relation between FMs and FUMs are given to stimulate discussion and continuative research projects.

### FUMs as Peramorphic FMs

Ongoing fractionation defining the transition from flowers to floral units may be based on a delay in the expression of floral organ identity genes.

• FMs can be defined as determinate reproductive meristems with an early expression of floral organ identity genes (Coen and Meyerowitz, 1991). Their determinancy is regulated by *LEAFY* (*LFY*) suppressing *TERMINAL FLOWER* (*TFL)* and thereby stimulating the expression of *APETALA 1 (AP1*) and other ABC-genes (Turck et al., 2008; Zeevaart, 2008). The meristem fractionates only once and gives rise to sepals, petals, stamens and carpels. If it expands, new space is generated among already existing floral organs as demonstrated by secondary polyandry (Ronse De Craene and Smets, 1991) or corona formation (Claßen-Bockhoff and Meyer, 2015).
• In FUMs, compared to FMs, the expression of floral identity genes starts later allowing the meristem to fractionate before each sub-meristem merges into a flower. Consequently, the result is not a single flower but a multi-flowered unit. As shown in *Euphorbia* (Prenner et al., 2011) and *Gerbera* (Zhao et al., 2016), *LFY*-like genes are expressed most likely defining the determinacy of their meristems in a similar way as in flower meristems. The repeated subdivisions would just be the autonomous response of the meristem to its ongoing expansion (Runions et al., 2014). The resulting fractionation pattern would reflect the geometry of the meristem which explains the repeated cross-wise splits in *Ricinus*. Fractionation only stops after five to six steps of subdivision when the latest sub-meristems are defined as flowers originating anthers.

If the delay of gene expression triggers the process of repeated fractionation, one may interpret the FUM as a peramorphic FM. The process of peramorphosis is defined as an extension of trait development, producing new morphologies in the descendant which are not present in the ancestors (Box and Glover, 2010). In the case of *Ricinus*, it occurs due to a prolongation of the not yet genetically defined stage of meristem development.

### Flower-Inflorescence Boundaries

FUMs also offer a new view onto the phenomenon of intermediate structures between flowers and inflorescences. From the evolutionary point of view, one should expect such forms as all types of reproductive meristems originate from the SAM and finally produce flowers.

While in the past, traditionally defined inflorescences (developing from inflorescence meristems) were used as reference systems demanding a deep re-organization of the inflorescence to generate a flower-like pattern (e.g., Harris, 1999), the use of FUMs easily explains flower-like units from flower-like meristems. Only single steps of fractionation separate a flower from a head and a head from a secondary head (Claßen-Bockhoff, 2016). This easy way to transform a meristem is supported by mutants of *Gerbera* (Asteraceae) illustrating that several flowers develop at a single ray flower position under certain genetic and spatial conditions (Zhao et al., 2016).

While the heads in Asteraceae are composed of clearly identifiable flowers, cyathia in *Euphorbia* show indistinct boundaries between floral organs, flowers and inflorescences. Prenner and Rudall (2007) concluded from their studies that the cyathium should be neither interpreted as a flower nor as an inflorescence, but as a “hybrid.” The lacking boundaries are excellent examples for the overlap of two independent processes, the development of the original meristem forming the cyathium and that of its parts. The higher the parts are reduced, the more difficult is the interpretation of the whole as a flower or floral unit as both meristems converge to a flower-like pattern. The extreme case is shown in the staminate unit of *R. communis*, in which the flowers are so extremely reduced that only repeated fractionations indicate the underlying complex nature.

The difficulty to identify flower-inflorescence boundaries may be due to the variability of the FUM in the number of fractionations. The labile balance between meristem expansion (stimulating fractionation) and the time of gene expression (defining flower meristems) may result in transitional forms. However, these are no hybrids but structures indicating a process of evolutionary character transformation.

## Data Availability Statement

The datasets generated for this study are available on request to the corresponding author.

## Author Contributions

RC-B initiated the project conducted by undergraduate students. RC-B and HF supervised the student projects. HF added missing SEM-pictures and histological cuttings. RC-B wrote the manuscript which was discussed with HF and revised by both authors.

## Conflict of Interest

The authors declare that the research was conducted in the absence of any commercial or financial relationships that could be construed as a potential conflict of interest.
